# 

**Published:** 1982

**Authors:** 


NATIONAL

LIBRARY

S 15 FEB 1983 Z

OF

MEDICINE

### BRISTOL MEDICO-CHIRURGICAL JOURNAL

JULY/OCTOBER 1982

VOLUME 97 iii/iv

